# Editorial: Prediction of severity of food allergy

**DOI:** 10.3389/falgy.2025.1717271

**Published:** 2025-11-06

**Authors:** Richard L. Wasserman

**Affiliations:** Pediatrics Department, Medical City Children’s Hospital, Dallas, TX, United States

**Keywords:** food allergy, anaphylaxis, basophil activation testing, component resolved diagnostics, multiplex allergy testing, mass spectroscopy, pollen counts

When a food allergy (FA) diagnosis is made, virtually every patient or parent asks, “How bad will a reaction be?” Whether the food allergy evaluation was motivated by an episode of anaphylaxis or an elevated specific IgE (sIgE) on a screening test, this question is part of every conversation. As with every allergic reaction, both the triggering food allergen and host factors play a crucial role in determining the answer to that question. Because food allergen exposures triggering a reaction are almost always accidental, the dose is rarely known before the reaction occurs. Understanding other factors related to the trigger, such as cooking, baking, and enzymatic treatment (yogurt or cheese), which denature the allergenic proteins, is seldom helpful because processing either eliminates allergenicity for the specific patient or has no effect. Similarly, identifying a reaction threshold dose provides a binary result: the patient will either react to a given dose or not, but it says nothing about the severity of the reaction should the threshold be exceeded. Other factors relating to the triggering food, such as the relative concentrations of allergenic proteins and isoforms of those proteins, are the subject of speculation but are not helpful in a clinical context. Oral food challenges, the diagnostic gold standard, identify the reaction-eliciting dose but are not designed to characterize reaction severity. Thus, host factors are the only clinically useful predictors of food allergy reaction severity.

The review by Fitzhugh provides a helpful overview of host-specific factors and analytic evaluations that inform the clinical discussion of reaction severity. Although most food allergy patients exhibit atopic co-morbidities, co-existent atopic diatheses do not predict FA reaction severity with the possible exception of asthma. The role of asthma is controversial (1, 2). Although a meta-analysis showed no relationship between asthma and severe reactions (3), Wasserman et al. (4) reported a multivariate analysis showing that while persistent asthma was not associated with peanut oral immunotherapy (OIT) outcome, the presence of intermittent asthma decreased the likelihood of reaching the target dose, suggesting that some persistent asthmatics were undertreated. Zhou et al. report on the relationship between mugwort sensitization and anaphylaxis provides contrasting data for both pollen allergy and asthma. They have shown a seasonal correlation between mugwort pollen counts and severe FA reactions (Zhou et al.). They also found an association of severe reactions with asthma. The truism that the severity of a reaction does not predict the severity of the next reaction works both ways; a history of anaphylaxis is a poor predictor of future anaphylaxis. Although intuitively, higher sIgE and skin prick tests (SPT) and low reaction thresholds should predict more severe reactions, there is no high-quality data supporting this concept. These parameters correlate well with the likelihood of a reaction but poorly with the severity of a reaction.

Age (1, 5) and risk-taking behavior on the part of adolescents and adults are associated with fatal FA reactions but may not be independent variables (6). We have learned from the OIT experience that exercise, alcohol consumption, certain medications, and viral infection lower the threshold for a FA reaction. However, these factors may not worsen the severity of those reactions. These limitations of host-related data mean that biomarkers offer the best hope of predicting reaction severity.

As noted above, high sIgE and SPT results correlate well with the likelihood of a reaction. This information can be further refined serologically using component resolved diagnostics and epitope-based assays. Component resolved testing for peanut can determine if a patient's antibodies are directed at PR-10 proteins or storage proteins, indicating a lower or higher risk for systemic reaction, respectively. Component resolved testing can also support the diagnosis of pollen food allergy syndrome, which poses a low risk of severe reactions. Epitope-based testing can provide additional information that identifies sensitization to primary amino acid sequences associated with reactivity, leading to the categorization of reactivity thresholds into high, medium, or low (7). The ability to efficiently test antibodies to multiple proteins/peptides is crucial to the value of serologic testing. Multiplex assays are often the most efficient approach to exploiting serology to manage food allergic patients. Depending, however, on the patient's food allergy history, singleplex testing to one or a few allergenic proteins is sufficient. Basophil Activation Testing (BAT), the *in vitro* correlate of clinical reactivity, has a higher positive and negative predictive value for reactivity than serological assays. Santos et al. have reported strong correlations between the magnitude of BAT reactivity and reaction threshold, suggesting that BAT may be a marker of reaction severity (8).

Several technical challenges limit the utility of basophil activation testing, including test accessibility, a challenging workflow, and a significant percentage of patients who do not respond to positive controls. Wheeler et al. provide a robust discussion of the virtues of mass spectrometry as a complement to BAT, which has the potential to ameliorate some of the limitations of BAT, thereby improving the utility of this potentially important modality.

Clinically useful *in vitro* assays must correlate with *in vivo* conditions in patients. Additionally, unless there is a single worldwide provider of the *in vitro* assay, all versions of the assay must be comparable. de Boer et al. report a study of multiplex assay design performance with particular attention to the issues of isoallergens and variants. They found considerable heterogeneity among the commercially available multiplex peanut allergy assays. These assays provided comparable results for Ara h storage proteins but not for the LTP Ara h9. This study highlights the importance of collaboration among multiplex allergy assay manufacturers, as well as the need for clinician education on what these assays actually measure. Although de Boer and colleagues evaluated multiplex assays, singleplex assays are subject to the same concerns relating to allergen heterogeneity and comparability of assays produced by different manufacturers.

In the future, clinicians will come to rely on both multiplex serologic testing and BAT to develop biomarkers of FA reaction severity. Physicians caring for patients with food allergies need to understand the strengths and limitations of both assays.
